# A comparison of coblation and modified monopolar tonsillectomy in adults

**DOI:** 10.1186/s12893-023-02035-1

**Published:** 2023-05-19

**Authors:** Zhengcai Lou

**Affiliations:** Department of operating theater, Yiwu central Hospital, 699 jiangdong road, 322000 Yiwu city, Zhejiang provice China

**Keywords:** Coblation, Electrocautery, Hemorrhage, Inferior pole, Pain scores, Tonsillectomy

## Abstract

**Objective:**

To compare the intraoperative records and postoperative clinical outcomes of adults who underwent coblation and modified monopolar tonsillectomy tonsillectomies.

**Materials and methods:**

Adult patients with tonsillectomy were randomly divided into the coblation and modified monopolar tonsillectomy groups. The estimated blood loss, postoperative pain score, operation time, post-tonsillectomy hemorrhage (PTH), and cost of disposable equipment were compared.

**Results:**

Pain intensity in the coblation and monopolar groups was similar on postoperative days 3 and 7. However, the mean maximum pain score in the monopolar group was significantly higher compared to the coblation group on postoperative days 1 (*P* < 0.01) and 2 (*P* < 0.05).Secondary PTH occurred in 7.1% (23/326) of patients in the coblation group and 2.8% (9/327) of patients in the monopolar group (*P* < 0.05).

**Conclusion:**

Although pain was significantly increased on postoperative days 1 and 2 in the modified monopolar tonsillectomy group, the operation time, secondary PTH, and medical costs were significantly decreased compared to the coblation technique group.

## Introduction

**Fig. 1:**
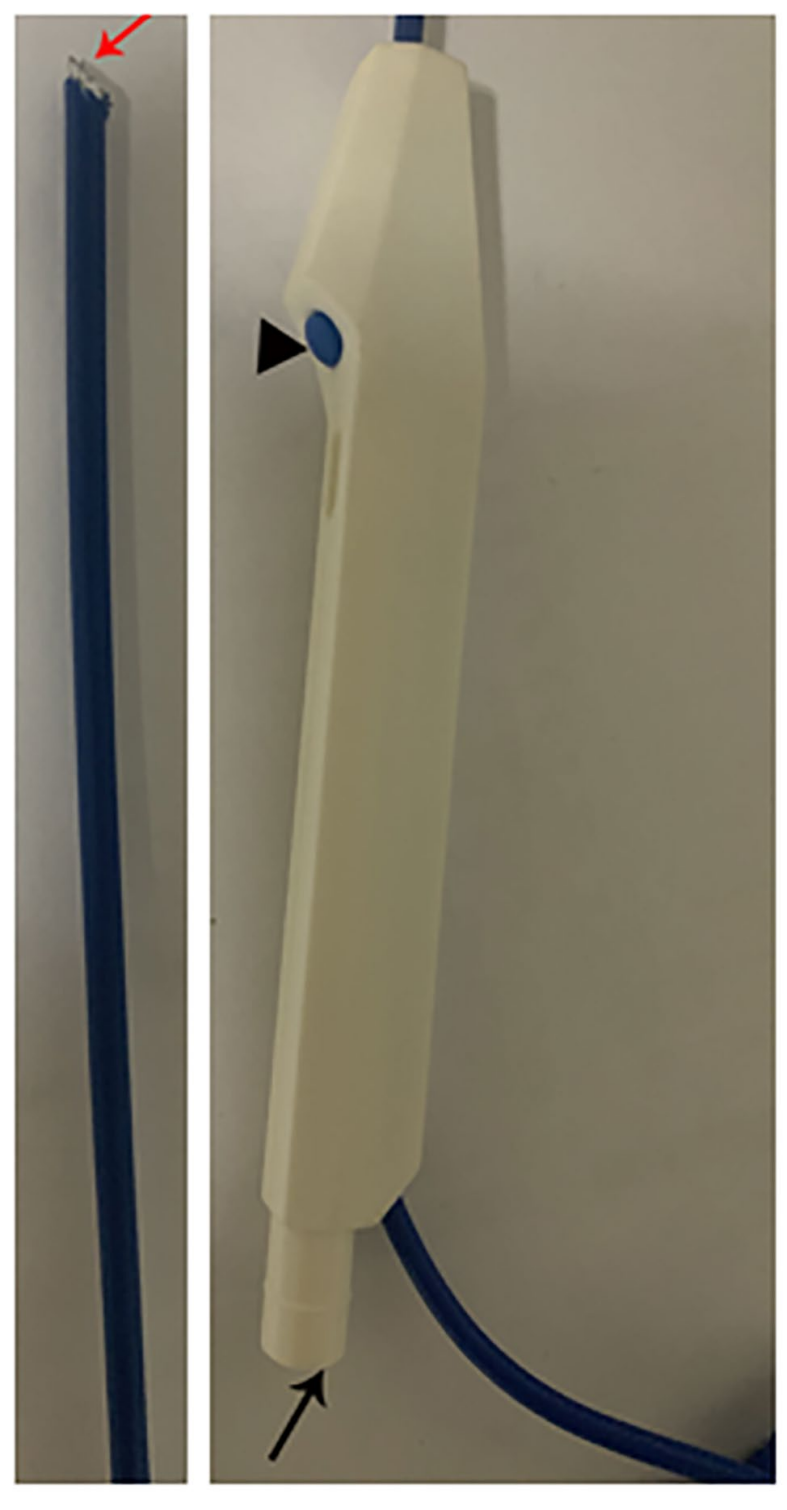
Modified monopolar device. Red arrow indicated needle-like front-end, black arrow indicated a suction hole, black triangle indicated the button

Tonsillectomy is used for the treatment of chronic tonsillitis, benign tonsillar masses, and obstructive sleep apnea hypopnea syndrome induced by tonsillar hypertrophy, and as a precursor surgery for styloidectomy. It is one of the most commonly performed surgeries in the otorhinolaryngologic department. Due to postoperative pain, hemorrhage, and long operation times, traditional cold steel dissection has gradually been replaced by coblation and electrocautery techniques.Unfortunately, no consensus exists regarding the best surgical technique for tonsillectomy [1, 2]. Although coblation tonsillectomy has the advantage of less postoperative pain, the cost of coblation is high [3, 4]. In recent years, we used a long handle monopolar device with a suction hole and the button, in which the monopolar front-end was modified to a needle-like shape, thereby formed a modified monopolar device **(**Fig. 1**)**, which was used to surgically dissect the tonsil. Modified monopolar coagulation have been used for tonsillectomies in our practice, with good outcomes achieved in adults. The aim of this study was to compare the intraoperative blood loss, postoperative pain, post-tonsillectomy hemorrhage (PTH), and medical costs associated with adult tonsillectomies between coblation and modified monopolar tonsillectomy methods.

## Materials and methods

### Ethical considerations

The study protocol was approved by the Institutional Ethical Review Board of Yiwu central Hospital, China. Informed consent was obtained from all participants.

### Methods

Adult patients who underwent tonsillectomy due to tonsillar hypertrophy.

or recurrent tonsillitis in the Department of Otorhinolaryngology-Head and Neck Surgery between April 2012 to September 2020 were recruited. The exclusion criteria were acute tonsillitis within 2 weeks, peritonsillitis or peritonsillar abscess, bleeding disorder, immunodeficiency syndrome, and systemic disease. A sample size calculation was performed using Power Analysis and Sample Size software (version 11.0). Our previous hemorrhage rate of two techniques are used to calculate the sample size, for an alpha level of 0.025, a desired type II error rate of 0.1, and power of 0.9. The total calculated sample size was 613. Therefore, 620 subjects were enrolled, affording adequate power in this study. Group allocation was performed by the principal investigator and a registered operating room nurse using simple random sampling. Specifically, consecutive subjects who met the inclusion criteria and signed the consent form were assigned random numbers generated by the SPSS for Windows software package (ver.18.0; SPSS, Inc., Chicago, IL, USA), which allocated them to either coblation or modified modified monopolar tonsillectomy group.All surgeries were performed by the senior author, who has more than 20 years of experience with tonsillectomy and 5 years of coblation. However, principal investigator didn’t perform the surgery for patients.

### **Surgical** technique

In all patients, the procedure was performed under general anesthesia. A Boyle–Davis mouth gag was used to stabilize the tracheal tube during surgery to ensure visualization of the oropharynx.

### **Identification** and **separation of the upper pole and middle portion of the tonsil**

**Fig. 2:**
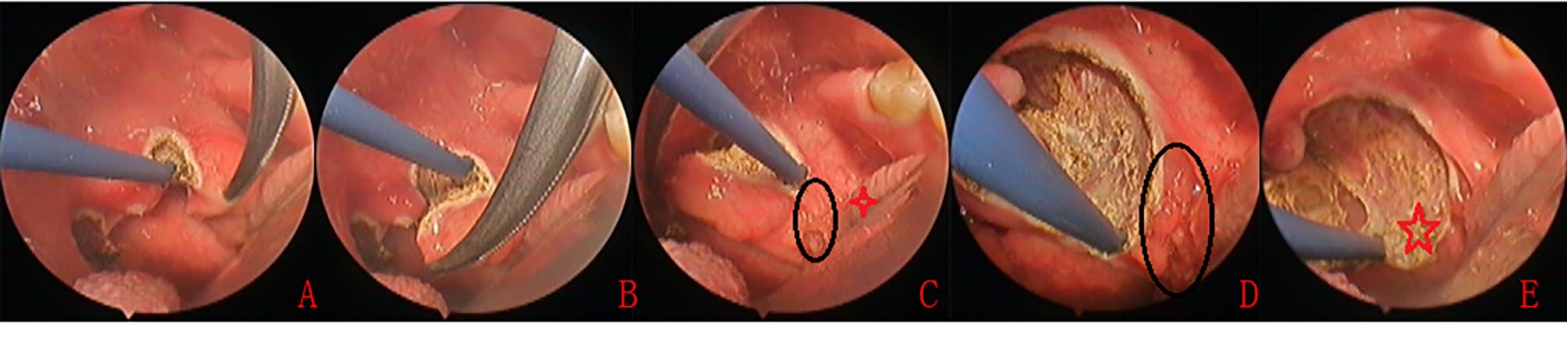
Monopolar technique. Identification and dissection of the upper pole (**A** and **B**). Dissection of the tonsillar tissue(**C**) and removal of inferior pole (**D**), tonsillar fossa (**E**). The black oval: dense connective tissue; four red stars:areas of the inferior pole

The triangular area above the upper pole of the tonsil was identified, and the upper pole was separated by digital palpation. The tonsillar tissue of the upper pole was grasped with small forceps and pulled medially and inferiorly to reveal the avascular space (i.e., peritonsillar space) above the tonsil (Fig. 2A and B). The tonsil was carefully dissected along the peritonsillar space with medial and inferior traction to avoid muscle penetration (Video 1).

### Dissection of the inferior pole

During dissection, traction was applied in the medial and superior directions to expose the inferior pole of the tonsil. The inferior pole capsule is linked to the lingual lymphoid tissue through a narrow and dense tract of fibrous connective tissue, which contains a rich arterial vessel network. Care should be taken to ensure that the pharyngeal constrictor muscle is not damaged during dissection of the inferior pole; specifically, the dissection should be limited to the inferior pole capsule to minimize the risk of injury to the narrow and dense fibrous connective tissue (Fig. 2C, D, and E indicated by the black oval and red star).

### Coblation technique

The Evac 70 coblation system (Smith & Nephew, London, UK) was used to surgically dissect the tonsil. During the procedure, the ablation power of the Evac 70 wand was set to 7, with a coagulation power of 3. Bipolar coagulation and vessel ligation were not performed.

### Modified monopolar tonsillectomy technique

We used a long handle monopolar device with a suction hole and the button, in which the monopolar front-end was modified to a needle-like shape, thereby formed a modified monopolar device (Fig. 1**)**, which was used to surgically dissect the tonsil.

The cautery was controlled by a switch on the handle, and the coagulation was set to 25 W(Fig. 2). Vessel ligation was not used.

### Follow-up

The patients received a liquid diet and intravenous antibiotics postoperatively, were discharged on the second day after surgery, and presented to the outpatient clinic for follow-up at postoperative day 7. Postoperative pain was evaluated by a senior surgeon who were blinded to treatment groups, in the early morning before breakfast using a visual analog scale (VAS) on postoperative days 1, 2, 3, and 7.The anesthesia, hospitalization, nursing, and medical costs were similar for both techniques. However, we only considered the cost of disposable equipment (coblation or monopolar coagulation) when calculating the medical costs. The operation time was calculated from successful exposure of the oropharynx using a mouth gag to complete removal of the bilateral palatine tonsils. Intraoperative blood loss was estimated by the surgeon. Postoperative hemorrhage was defined as bleeding of any severity that required surgery. Primary PTH was defined as postoperative hemorrhage within 24 h of surgery, and secondary PTH as that occurring after the first 24 h. All parents were advised to return to the outpatient department for a formal follow-up.

### Statistical analysis

Statistical analyses were performed using SPSS software (version 18.0; SPSS Inc., Chicago, IL, USA). Categorical variables are reported as frequencies and percentages, and continuous variables as means (standard deviation [SD]) or medians. Pearson chi-squared and t-test were used to compare groups. P-values < 0.05 were considered statistically significant.

## Results

This study enrolled 653 patients (coblation group, n = 326; monopolar group, n = 327), including 383 male and 270 female. The average age of the patients was 30.9 ± 6.8 (range: 18–46) years. Age, sex, disease duration, and surgical indications were similar for both groups (Table 1).

All patients completed 7 days of follow-up. Pain intensity in the coblation and monopolar groups was similar on postoperative days 3 and 7 (Table 1). However, the mean maximum pain scores differed significantly between the two groups on postoperative days 1 (*P* < 0.01) and 2 (*P* < 0.05). Uvula-significant edema was observed in 39.3% (46/117) of the coblation group and 85.4% (129/151) of the monopolar group (*P* < 0.01). Table 2 shows that the mean operation time was significantly shorter in the monopolar group compared to the coblation group (17.4 ± 4.8 vs. 28.6 ± 3.3 min,*P* < 0.01). Intraoperative estimated blood loss did differ significantly between the groups (*P* < 0.01) (Table 3). The cost of the disposable equipment was US$ 430.48 and US$ 28.18 in the coblation and monopolar groups, respectively (*P* < 0.01). No instrument-related complications were observed in either group.

PTH occurred in 46 patients (7.04%) and required additional surgery, the bleeding site was in the lower pole in 40 (87.0%) and in the middle portion in 6 (13.0%), primary PTH was in 30.4% (14/46) patients and secondary PTH in 69.6% (32/46). However, PTH occurred in 9.8% (32/326) patients in the coblation group (including 2.8% in primary PTH and 7.1% in secondary PTH) and 4.3% (14/327) patients in the monopolar group (including 1.5% in primary PTH and 2.8% in secondary PTH).

Of the 46 patients with PTH, the lymphoid tissue of lingual extension was damaged in 86.9% (40/46) patients. In addition, of the 32 patients with PTH in the coblation group, 25 (78.1%) and 7 (21.9%) required second and third surgeries, respectively; while all the 14 patients in the monopolar group required a second surgery.

## Discussion

Although tonsillectomy is one of the most widely conducted otolaryngologic surgeries, there is no universally accepted consensus regarding the use of surgical instruments [5–7]. This study showed that intraoperative estimated blood loss did not differ significantly between the coblation group and modified electrocautery groups, whereas the mean operation time was significantly shorter in the latter group. These findings were similar to those of previous studies. Several studies have shown that coblation takes significantly longer to complete than electrocautery [8, 9]. Noordzij et al. [9] reported that the mean time to remove a single tonsil with coblation and electrocautery was 8.22 and 6.33 min, respectively (p = 0.011). Intraoperative blood loss and the operation time depend on the clinician’s skill in most cases. We observed that intraoperative bleeding was typically due to damage of the blood capillaries in the pharyngeal muscles. Less intraoperative bleeding is likely only if the tonsil is dissected within the peritonsillar space, because the peritonsillar space is avascular. Most adult tonsillectomy patients also experience tonsillar hypertrophy and chronic inflammation, and have difficult oropharyngeal exposure and scar tissue hyperplasia. The large outer diameter and lower temperature of the coblator wand (40–60 °C) not only affects the operative field exposure, but also the ability to cut the scar tissue. However, the needle tip bovie of the modified modified monopolar tonsillectomy device do not affect the operative field; additionally, the high temperature of the device (> 400 °C) enables rapid dissection of the scar tissue of the peritonsillar space, thereby shortening the operation time.

The preference for the coblation technique over other methods of some clinicians is due to the minimal damage to the surrounding tissue and lower postoperative pain score [1, 10]. However, no significant difference in mean pain scores was found between our coblation and monopolar groups on postoperative day 3 or 7, although there were differences on postoperative days 1 and 2. This finding was similar to those of previous studies. Most of which reported no advantage of the coblation technique with respect to the pain scores of adults [7, 8, 11, 12]. Álvarez Palacios et al. [11] compared the postoperative pain associated with three techniques (cold dissection, monopolar-bipolar dissection, and coblation dissection) in 103 elective adult patients undergoing tonsillectomy; no significant difference was found in the post-tonsillectomy pain scores among the techniques. Hong et al. [12] also found no significant difference in postoperative pain between coblation and electrocautery tonsillectomies in adults; they suggested that coblation tonsillectomy warrants further study with respect to the potential for less postoperative pain. Additionally, some studies reported no correlation between surgical technique and pain score, and that the overall outcomes did not favor coblation over bipolar techniques [13, 14]. Surprisingly, Hasan et al. [14] reported that their coblation group showed higher pain scores 1 and 3 h postoperatively, and a higher number of analgesic doses, compared to those undergoing bipolar tonsillectomy. We speculate that the discordance in postoperative pain assessments may be explained by differences in patient age, pain tolerance, and postoperative pain assessment timepoints. Usually, longer-duration inflammation coincides with a greater amount of scar tissue and more significant postoperative pain in older patients. There is little doubt that uvula edema in our monopolar group was more significant compared to the coblation group, and could be a factor in the early postoperative pain seen in the former group.

PTH requiring surgery is a significant complication following tonsillectomy; it not only increases secondary medical costs, but also anxiety for patients and their families. The incidence rate of PTH requiring surgery was 7.04% in this study, consistent with the rates of 1.4–11.9% in previous reports [15–18]. However, our results are inconsistent with previous studies with respect to the technology and procedures used for PTH [8, 19, 20]. Most studies reported that coblation tonsillectomy was associated with a higher incidence of secondary hemorrhage [17, 21–23]. Noon et al. [18] compared the rate of PTH between coblation tonsillectomy and dissection with bipolar coagulation in 65 adults; the rate was significantly higher in the former group (22.2% vs. 3.4%) and coblation was subsequently discontinued in their department. Lane et al. [24] reported an association between the PTH rate and instrumentation, with more PTH cases involving coblation (58.9%) than electrocautery (23%). Lowe and Van der Meulen found that the PTH rate with coblation was 3.4 times higher compared to that after cold steel excision/dissection [25]. In the current study, although there was not a significant difference in the incidence primary PTH between the two groups, the incidence of secondary PTH in the coblation group was significantly higher than that in the monopolar group. We speculate that the results could be related to differences in the instrumentation used. A needle tip bovie of modified modified monopolar tonsillectomy could improve precision of cautery.

In this study, the lower pole and middle portion were the sites of bleeding in 40 (87.0%) and 6 (13.0%) of the 46 patients with PTH, respectively. We found that the dense fibrous connective tissue between the tonsillar capsule and lingual extensions of the lymphoid tissue in the inferior pole was damaged in 40 patients with PTH, in which the dissection extended beyond the fibrous connective tissue containing rich arterial blood vessels [26]. Thus, some researchers have suggested suture of the inferior pole [23, 27], while others prefer lower pole capsule preservation [26] to reduce PTH. We believe that PTH of the inferior pole is associated with instrument performance. For small blood vessels, closure by operation in ablation mode may be sufficient, while for medium- or large-diameter vessels, this closure is unreliable and increases the risk of secondary hemorrhage. However, such vessels can be effectively coagulated by modified monopolar tonsillectomy due to the high temperature. Thus, accurate identification of the lower pole capsule and dense fibrous connective tissue is crucial, and dissection of the inferior pole should not extend beyond the connective tissue boundary. In addition, modified modified monopolar tonsillectomy has significantly lower medical costs compared to the coblation technique, and is thus preferred in economically underdeveloped areas. [3, 28]. Sakki et al. [29] suggested in a recent pediatric tonsillectomy study that monopolar electrosurgery be used because it is a simple procedure and the monopolar device is readily available in every operating room. Given the findings of our study, of no significant differences in pain between the two techniques, but a considerable difference in cost, or operation time, and secondary PTH, we question whether coblation is necessary.

## Conclusions

Although modified monopolar tonsillectomy group significantly increased the pain on postoperative days 1 and 2, it significantly reduced the mean operation time, secondary PTH, and medical costs compared to the coblation technique group. In addition, for prevention of PTH, dissection of the tonsil should not extend beyond the dense fibrous connective tissue between the inferior pole capsule and lingua.

**Table 1:** Demographics,operation time, pain scores,blood loss,blood loss, and cost of two groups

	Coblation group (n = 326)	Monopolar group (n = 327)	*t or X*^*2*^ value	P value
Age	29.3 ± 3.7	31.8 ± 2.6	0.546	0.699
Sex (female: male)	129:197	141:186	0.428	0.400
Duration, years	10.3 ± 3.1	11.2 ± 1.9	0.189	0.396
Indication (chronic tonsillitis: tonsillar hypertrophy)	116:210	125:202	0.382	0.536
Operation time, minutes	28.6 ± 3.3	17.4 ± 4.8	42.371	< 0.01*
Intraoperative estimated blood loss, ml	8.1 ± 3.6	6.3 ± 2.7	13.491	< 0.01*
Postoperative pain scores				
Day 1	4.7 ± 3.1	7.1 ± 2.2	28.132	< 0.01*
Day 2	4.1 ± 1.6	5.2 ± 1.7	4.871	< 0.05*
Day 3	3.3 ± 1.5	3.9 ± 1.2	0.327	0.571
Day 7	2.8 ± 1.2	3.0 ± 1.5	0.543	0.849
Primary hemorrhage, n(%)	9 (2.8)	5 (1.5)	0.667	0.414
Secondary hemorrhage, n(%)	23 (7.1)	9 (2.8)	5.595	< 0.05*
Cost of disposable equipment,US$	430.48	28.18	17.216	< 0.01*

**Table 2:** The percentage of operation time of two groups

	< 10 min	11−20 min	21−30 min	> 30 min	*X* ^*2*^	*P*
Coblation group (n = 326)	0 (0.0%)	121 (37.1%)	175 (53.7%)	30 (9.2%)	46.06	< 0.01
Monopolar group (n = 327)	45 (13.8%)	182 (55.6%)	87 (26.6%)	13 (4.0%)		

**Table 3:** The percentage of estimated blood loss of two groups

	< 5ml	5−20ml	21–50	*X* ^*2*^	*P*
Coblation group (n = 326)	226 (69.2%)	67 (20.5%)	34 (10.3%)	14.42	< 0.01
Monopolar group (n = 327)	273 (83.4%)	45 (13.9%)	9 (2.6%)		

The English in this document has been checked by at least two professional editors, both native speakers of English. For a certificate, please see:

http://www.textcheck.com/certificate/aWqOjO

## Data Availability

All data generated or analyzed during this study are included in the published article.

## Declarations

**Ethics approval and consent to participate**.

The study protocol was approved by the Human Research Ethics Committee of Yiwu Central Hospital, which adheres to local policy, national laws, and the World Medical Association Declaration of Helsinki. Written informed consent was obtained from all patients and also from parents of the participants.

A statement to confirm that all methods were carried out in accordance with relevant guidelines and regulations.

**Consent for publicatio**n.
